# On Incipient Plasticity of InP Crystal: A Molecular Dynamics Study

**DOI:** 10.3390/ma14154157

**Published:** 2021-07-26

**Authors:** Dariusz Chrobak, Grzegorz Ziółkowski, Artur Chrobak

**Affiliations:** 1 Institute of Materials Engineering, University of Silesia in Katowice, 75 Pułku Piechoty 1A, 41-500 Chorzów, Poland; 2 Institute of Physics, University of Silesia in Katowice, 75 Pułku Piechoty 1A, 41-500 Chorzów, Poland; grzegorz.ziolkowski@us.edu.pl (G.Z.); artur.chrobak@us.edu.pl (A.C.)

**Keywords:** InP, incipient plasticity, phase transformation, molecular dynamics

## Abstract

With classical molecular dynamics simulations, we demonstrated that doping of the InP crystal with Zn and S atoms reduces the pressure of the B3→B1 phase transformation as well as inhibits the development of a dislocation structure. On this basis, we propose a method for determining the phenomenon that initiates nanoscale plasticity in semiconductors. When applied to the outcomes of nanoindentation experiments, it predicts the dislocation origin of the elastic-plastic transition in InP crystal and the phase transformation origin of GaAs incipient plasticity.

## 1. Introduction

Recently, Wong et al. [1] used nanoindentation to create a stable, metallic nanoparticle at the surface of Si crystal. This nanostructure can be thought of as an efficient light-catching system with promising applications in photovoltaic technology [2,3]. Unexpectedly, the authors breathed new life into the importance of research of phenomena that can initiate nanoscale plasticity in semiconductor crystals. Indeed, it is not possible to create metallic nanoislands by means of nanoindentation in all semiconductors, but rather only in those whose plastic deformation is initiated by the transformation to the high-pressure metallic phase.

On the whole, elastic-plastic transition in dislocation-free semiconductor nanovolume is a controversial issue, as it involves both the phase transformations and dislocation generation. For example, nanoindentation-induced plasticity of Si crystal can be initiated by phase transformation or nucleation of dislocations, depending on experimental conditions [1,4,5]. Interestingly, the results of nanoindentations accompanied by Raman spectroscopy [6,7,8] indicate a complex course of phase transformations in silicon: formation of Si-II (β-tin) phase during loading and Si-III (bc8) and Si-XII (r8) phases during unloading. Regarding other semiconductors, it is worth noting that the elastic-plastic transition in GaAs crystal is initiated by the B3→Cmcm phase transformation [9,10,11], while nanoscale plasticity in GaN is exclusively governed by the activity of dislocations [12,13].

Determination of the phenomenon which initiates nanoindentation-induced elastic-plastic transition seems to be a difficult task due to the fact that crystal deformation occurs in very small volumes. One of the methods that can be used involves disturbing the structure of a semiconductor by introducing dopant atoms and then examining how the doping affects the contact pressure during the elastic-plastic transition. To be more precise, we will focus on theoretical demonstration and experimental verification of how doping influences the elastic-plastic transition in one of the most important semiconductors in technology, namely InP. A review of the scientific literature shows that the optoelectronic properties of InP are basically known, while the cause of its elastic-plastic transition has not yet been fully determined. Looking at the available data, the dominant role of dislocation activity becomes clear in the plastic deformation of InP [14,15,16,17,18,19,20,21], without the participation of structural phase transformations [18]. However, the results of mentioned structural investigations were carried out after nanoindentation experiments, so it is premature to exclude the effect of phase transformation on the incipient plasticity of InP.

In this letter, classical molecular dynamic (MD) simulations were used to demonstrate the effect of Zn- as well as S-doping on both the transformation from B3 (zincblende) to B1 (rocksalt) crystal structure of InP and dislocation generation during nanoindentation of (001) the InP surface. The results of MD simulations allowed us to propose a method to distinguish the origin of the incipient plasticity of semiconductors.

## 2. Materials and Methods

Molecular dynamics simulations were performed with the LAMMPS software [22]. In order to study the effect of Zn- and S-doping on the pressure of B3→B1 phase transformation in InP crystal, the system (supercell) composed of 8×8×8 B3 unit cells (lattice constant $a_{B3}=5.869$ Å [23]) was used. Doping with Zn and S atoms was chosen because it is technologically important giving p-type and n-type electrical conductivity, respectively. Moreover, the dopant atoms build into the InP crystal lattice and create relatively simple substitutional point defects: ${\mathrm{Zn}}_{\mathrm{In}}$, $S_{P}$ [24]. In our simulations, the defect $Y_{X}$ was defined by the replacement of one X atom of the supercell by atom Y, which resulted in reasonable $9.7\times 10^{+18}\;{\mathrm{cm}}^{-3}$ concentration of dopants. Interatomic interactions in pure InP crystal were described by the Vashishta potential [25,26], while interactions with Zn and S atoms were modeled by the Lennard-Jones potential (refer to Supplementary Materials). The Velocity-Verlet time integration algorithm, with an increment of 2fs, was used through the simulations, while the canonical (NVT) ensemble was employed to control the thermodynamical variables. Prior to running deformation simulations, the system was relaxed to a thermal equilibrium at the target temperature of 300K. The volume of the supercell was then reduced by varying the length of its edges up to 10% in 100,000 time steps. Structural changes, recorded every 500 time steps, were analyzed using the OVITO software [27].

Nanoindentations of the B3 phase of InP were simulated at 300K. A block of InP crystal ($41.1\times 41.1\times 28.8\;{\mathrm{nm}}^{3}$, 1,930,600 atoms) was indented by a rigid sphere (R=12.9nm) in the (001) surface. The interactions between the diamond indenter tip and InP crystal were modeled by the repulsive term of the Buckingham potential with 4 Å cut-off radius. Nanodeformation was accomplished in a quasi-static manner by a sequence of indenter’s displacements with an increment of 0.5 Å every 15ps.

Examples of Lammps scripts implementing the above simulations can be found in Supplementary Materials.

## 3. Results and Discussion

**Figure 1:**
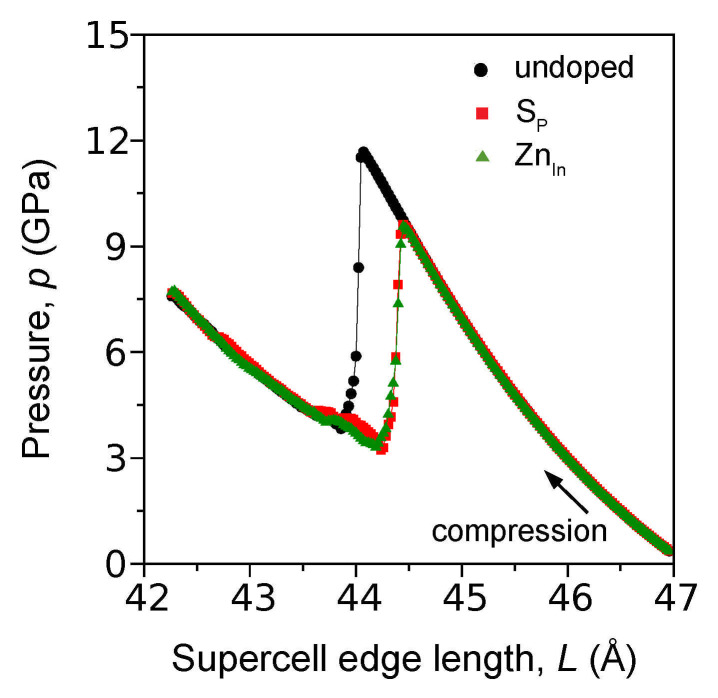
Results of MD simulations showing pressure (*p*) versus supercell edge length (*L*) relationship obtained for undoped as well as Zn- and S-doped InP crystal. The sudden drop of the pressure indicates instability and transformation of the initial B3 phase to a high-pressure one. Doping by Zn or S decreases the pressure of the phase transformation.

**Figure 2:**
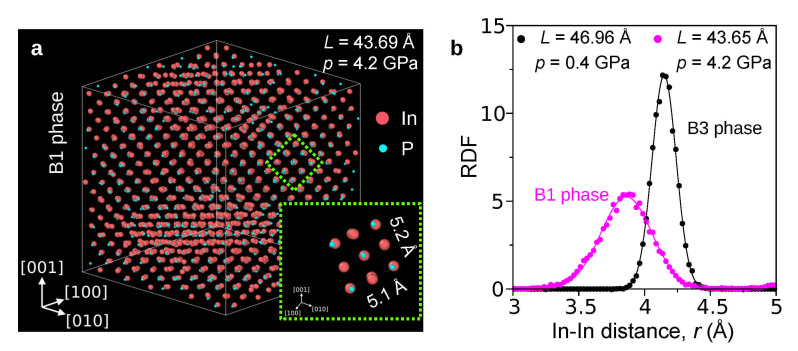
Results of MD simulations presenting details of the high-pressure phase of undoped InP. (**a**) Inspection of the supercell right after the phase transformation shows atomic arrangement of the high-pressure phase. Visualization of the unit cell of the high-pressure phase proves that it possesses the B1 structure; (**b**) radial distribution function (RDF) calculated for In-In bonds. Location of the RDF peak facilitates estimation of the mean value of both B3 and B1 lattice constants.

To investigate the effect of doping on the pressure of the B3→B1 phase transformation in InP, a supercell consisting of 8×8×8 unit cells ($a_{B3}=5.869$ Å) was used. As the initial edge length L=46.95 Å of the supercell gradually decreases (compression), the B3 phase of undoped InP becomes unstable at L=44.07 Å when the pressure *p* approaches the value $p_{PT}=11.7\;\mathrm{GPa}$ (Figure 1). A further decrease in *L* reduces the pressure to the level of ∼4 GPa, indicating formation of some high-pressure phase. The atomic configuration presented in Figure 2a (L=43.69 Å, p=4.2GPa) exhibits details characteristic for the B1 lattice. Indeed, the group of atoms shown in Figure 2a, cut from the column surrounded by the green dashed line, undoubtedly forms the B1 unit cell (see also Figure S1 in Supplementary Materials). In order to estimate the lattice constant of the B1 phase, we used the method based on the radial distribution function (RDF). Figure 2b shows RDFs calculated for the nearest In atoms in B3 (L=46.95 Å, p=0.4GPa) as well as B1 structure (L=43.69 Å, p=4.2GPa). It is clear that the mean value of the In-In distance (*d_In-In_*) decreases from 4.15 Å to 3.87 Å during the phase transformation. Finally, the mean value of the B1 lattice constant ($a_{B1}$ = 2*d_In-In_*/$\sqrt{2}$) is equal to 5.47 Å. On this basis, it can be concluded that the interatomic potential used by us models the B3→B1 phase transformation, and the obtained transformation pressure $p_{PT}=11.7\;\mathrm{GPa}$ agrees well with the literature data: $p_{PT}$ = 9.8–13 GPa [28,29,30].

Influence of ${\mathrm{Zn}}_{\mathrm{In}}$ and $S_{P}$ point defects on the structure of the B3 phase was first tested within the frame of DFT-based (Density Functional Theory) ab initio calculations (refer to Supplementary Materials). Substitution of Zn for the In atom creates Zn-P bonds characterized by the length $d_{Zn-P}=2.4151$ Å, which is less than the In-P bond length in the undoped crystal : $d_{In-P}=2.5747$ Å (contraction: −0.062%). In contrast, substitution of S for the P atom gives the bond In-S with the length $d_{In-S}=2.6644$ Å (expansion: +0.039%). Based on these data, the parameters of the Lennard-Jones potential used for description of the interaction between the dopants and the atoms of InP crystal were determined (see Supplementary Materials). Despite the local strain sign, the presence of ${\mathrm{Zn}}_{\mathrm{In}}$ and $S_{P}$ point defects violates the order of the B3 lattice and decreases the pressure of the B3→B1 phase transformation (Figure 1 as well as Figure S2 in Supplementary Materials). The difference in the transformation pressure caused by the point defects is very small; therefore, we assumed a common value for the pressure of the B3→B1 transformation in Zn- as well as S-doped InP crystal: $p_{PT}=9.6\;\mathrm{GPa}$ (L=44.47 Å). The modeled decrease of the B3→B1 phase transformation pressure, from 11.7GPa to 9.6GPa, corresponds with the results of X-ray diffraction experiments accompanied by the Raman spectroscopy carried out for Fe-doped InP [31].

**Figure 3:**
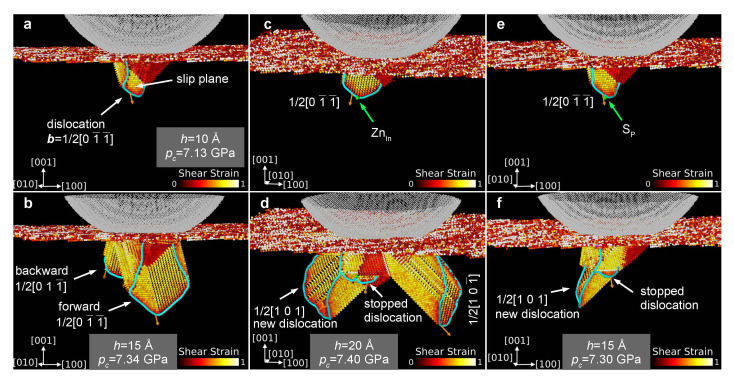
Example results of MD simulations showing the effect of point defects on the development of dislocations during nanoindentation of the InP (001) surface. (**a**,**b**) Undoped InP: as indentation proceeds, the initial dislocation with the Burger vector $b=1/2[0\bar{1}\bar{1}]$ develops and another dislocation (backward, $b=1/2[01\bar{1}]$) is generated. (**c**,**d**) Zn-doped InP: the presence of Zn atom in front of the initial dislocation ($b=1/2[0\bar{1}\bar{1}]$) stops its growth (well visible bending, pinning effect) and a new dislocations (b=1/2[101], $b=1/2[10\bar{1}]$) appear in the crystal. (**e**,**f**) S-doped InP: the presence of S atom acts in similar way, as in the case of Zn-doping. As a result, the development of existing dislocations is stopped. The increasing load can be accommodated by the emergence of new defects. The symbols *h* and $p_{c}$ denote the indentation depth and the instantaneous contact pressure value, respectively.

The effect of ${\mathrm{Zn}}_{\mathrm{In}}$ and $S_{P}$ point defects on the generation of dislocations during nanoindentation of (001) surface of InP can be demonstrated on the background of MD simulations performed for undoped crystal. Plastic deformation-preceded by formation of characteristic V-shaped shear-bands (see Figure S3 in Supplementary Materials)-starts at indentation depth h=10 Å (contact pressure $p_{c}=7.13\;\mathrm{GPa}$) when the first perfect dislocation (Figure 3a), characterized by the Burgers vector $b=1/2[0\bar{1}\bar{1}]$, appears beneath the indenter. Further increase of the indentation depth up to h=15 Å ($p_{c}=7.34\;\mathrm{GPa}$) results in development of the first perfect dislocation and generation of the second with a Burgers vector of $b=1/2[01\bar{1}]$, as presented in Figure 3b.

To show the impact of ${\mathrm{Zn}}_{\mathrm{In}}$ and $S_{P}$ point defects on the generation of dislocations, the simulations conducted for undoped InP were stopped when the indentation depth *h* reached the value of 10 Å and then the Zn or S atom was inserted into the place occupied by the In or P atom, respectively (Figure S4 in Supplementary Materials). The substitution was made on the slip plane of the first dislocation ($b=1/2[0\bar{1}\bar{1}]$) but slightly further towards its development direction (Figure 3c,e and Figure S4 in Supplementary Materials). After that, the simulations were continued, and it turned out that the growth of the first dislocation ($b=1/2[0\bar{1}\bar{1}]$) was stopped (pinning effect). Instead of its further development, an increase of the stress led to the creation of new dislocations with a Burgers vector of b=1/2[101] and $b=a/2[10\bar{1}]$ (Figure 3d,f). This result demonstrates the phenomenon suggested by a result of the literature data analysis, which indicates that doping the InP causes an increase in microhardness [32] as well as suppression of the dislocation generation during crystal growth [33,34]. The effect of doping on the development of dislocation structure is of stochastic nature, due to the thermal randomness of both the generation of dislocations and the location of point defects in a crystal lattice. However, when performing experiments with the nanoindentation method, it can be expected that the doping of the tested crystal will cause an increase in the mean value of the contact pressure at the beginning of plastic deformation, provided that the phenomenon initiating plasticity is dislocation nucleation. Indeed, as our simulations have shown, a point defect lying in the slip plane may stop the movement of dislocations and overcoming this obstacle or nucleating new dislocations will require an increase in stress.

The outcomes of our MD simulation made it possible to propose a method to determine the mechanism of nanoindentation-induced plastic deformation in InP. If the elastic-plastic transition is caused by the generation of dislocations, it could be expected that the doping increases the mean contact pressure at the onset of the plastic deformation. Moreover, if the elastic-plastic transition is caused by the phase transformation, the mean contact pressure should decrease due to doping.

**Figure 4:**
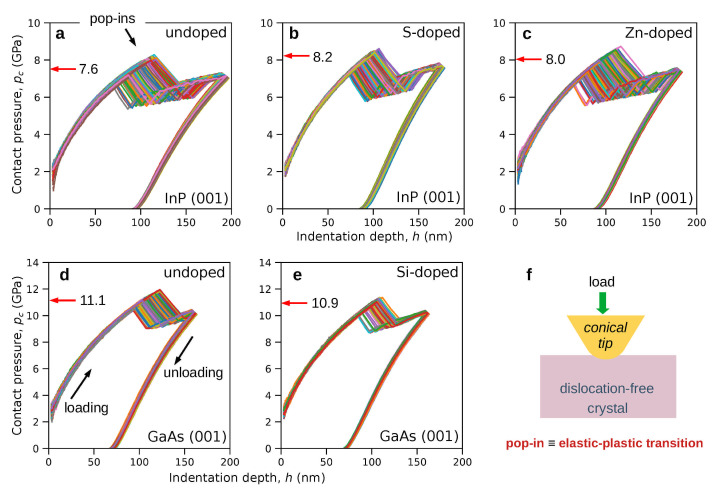
Results of nanoindentation experiments on the (001) InP surface: $p_{c}-h$ curves obtained for undoped (**a**) S- (**b**) and Zn-doped (**c**) crystals [14]. The red arrow assisted by the number indicates the mean contact pressure. Doping of InP shifts the mean contact pressure distribution towards higher values. In contrast, Si-doping of GaAs causes a decrease in the contact pressure (**d**,**e**). The last panel (**f**) presents a schematic of a nanoindentation experiment.

To demonstrate the usefulness of the proposed method, we recall the results of nanoindentation experiments [14] on Zn- and S-doped InP crystals characterized by the dopant concentrations $n_{Zn}=3.1-3.4\times 10^{+18}$ and $n_{S}=1.7-1.9\times 10^{+18}$, respectively. For the sake of comparison, we also investigated Si-doped GaAs ($n_{Si}=7.1-11.7\times 10^{+17}$), as it was already proved that the nanoindentation induced plasticity of GaAs starts from structural phase transformation (B3→Cmcm) [9,10,11]. We recorded numerous (∼400 for each sample, Figure 4) load (*P*) versus displacement (*h*) curves to determine the mean contact pressure at the elastic-plastic transition marked by the pop-in (discontinuity of the P(h) curve, details in Supplementary Materials). There was a significant shift of the contact pressure distributions recorded for InP towards higher values. The mean contact pressure at the onset of the elastic-plastic transition (pop-in) registered for undoped InP was 7.6GPa with standard deviation 0.4GPa (Figure 4a), while the values of pop-in measured for Zn and S doped crystal approached the values of 8.0±0.4GPa and 8.2±0.3GPa, respectively (Figure 4b,c). In light of our MD simulation, an increase in pop-in contact pressure indicates that the nanoindentation induced elastic-plastic transition in InP crystal is initiated by nucleation of dislocation. This is in contrast with the case of GaAs, for which a decrease of pop-in from 11.1±0.4GPa to 10.9±0.2GPa, caused by Si-doping, was measured (Figure 4d,e). In consequence, the nanoindentation induced elastic-plastic transition in GaAs is caused by the structural phase transformation.

## 4. Conclusions

In summary, we used MD simulations to study the effect of doping on the pressure of B3→B1 phase transformation and dislocation generation in nanoindented InP. It was shown that the presence of impurities in the crystal lattice reduces the pressure of phase transformation and inhibits the development of the dislocation structure. Based on these results, we propose a simple method that allows for distinguishing the phenomenon that initiates nanoscale plasticity in semiconductor crystals. It indicates that the nanoindentation induced plasticity InP is initiated by the generation of dislocations, while the nanoscale plasticity of GaAs begins the structural phase transition. By showing that the method works for both InP and GaAs, we expect that application of the method to other semiconductors will allow for investigating the nature of their nanoscale plasticity.

## Data Availability

The data presented in this study are available on request from the corresponding author.

## Supplementary Materials

Supplementary data and figures are available online at https://www.mdpi.com/article/10.3390/ma14154157/s1.
