# Supplementary material for: On Incipient Plasticity of InP Crystal: A Molecular Dynamics Study
Source: Materials (Basel). 2021 Jul 26;14(15):4157. doi: 10.3390/ma14154157 (PMC8348960; doi:10.3390/ma14154157)
Supplement: Supplementary file 1 [file materials-14-04157-s001.zip › SM_Chrobak-new.pdf]

Supplementary Materials

for

Incipient plasticity of InP crystal: A molecular dynamics study

Dariusz Chrobak[1], Grzegorz Ziółkowski[2], Artur Chrobak[2]

[1] Institute of Materials Engineering, University of Silesia in Katowice, 75 Pułku Piechoty 1 A, 41-500 Chorzów, Poland

[2] Institute of Physics, University of Silesia in Katowice, 75 Pu lku Piechoty 1 A, 41-500 Chorzów, Poland

1. Details of *ab-initio* calculations.

2. Parameters of Lennard-Jones potential for Zn and S dopant.

3. Supplemental figures.

4. Details of nanoindentation experiments.

5. Lammps scripts.

6. References.

1. *Ab-initio* calculations.

The purpose of the *ab initio* calculations was to obtain the data necessary to determine the parameters of the Lennard-Jones potential to describe the interaction of dopant atoms with the InP crystal atoms.

The *ab initio* simulations were performed with the Quantum Espresso code [1,2]. The ultrasoft pseudopotentials and Perdew-Burke-Ernzerhof exchange-correlation functional were used [3]. In order to achieve highly accurate calculations, the states of valence electrons were expanded into a series of plane waves with a kinetic energy cut-off of 40 Ry. Furthermore, the first Brillouin zone was sampled with the 5×5×5 Monkhorst-Pack mesh [4]. The effect of doping on structure of B3 phase of InP was studied using a supercell composed of 2×2×2 cubic unit cells. The central atom, P or In, of the supercell was replaced by an admixture: S or Zn atom, respectively. Furthermore, the relaxation of atomic positions was carried out until the interatomic forces were less than 1.0×10^{-5} Ry/a.u.^{3}.

2. Parameters of Lennard-Jones potential for Zn and S dopant.

The molecular dynamics simulations presented in the paper required the use of a specific model of interaction between the InP crystal atoms and the dopants: Zn and S. We decided to use the spherically symmetric Lennard-Jones potential

$$V_{LJ}(r) =- 4\varepsilon\left[\left(\frac{\sigma}{r}\right)^{12} - \left(\frac{\sigma}{r}\right)^{6}\right]$$

where the parameters (ε, σ) were fitted to reproduce data obtained from *ab initio* simulations.

First of all, we were interested in structural changes in nearest vicinity of the created point defect, namely in distances between the dopant (Zn, S) and surrounding atoms of the host crystal. Table I shows the results of the *ab initio* calculations compared with those obtained from MD simulations, which were carried out with the Lennard-Jones potential. The local atomic arrangement around the substitutional point defects are be modeled correctly with the selected parameter of the Lennard-Jones potential.

Table I Changes in local atomic environment caused by the presence of point defect. The symbols d and d_0 = 2.5412 Å refer to *ab initio* calculated distances between nearest neighboring atoms in doped and undoped InP crystal, respectively. Similarly, d_{MD} and $d_{MD,0}$ = 2.5418 Å mean interatomic distances obtained from MD simulations. The ε and σ parameters define the Lennard-Jones potential, which describes interactions between Zn and P atom as well as S and In atom.

	d (Å)	$(d-d_0)/d_0$	ε (eV)	σ (Å)	d_{MD} (Å)	$(d_{MD}-d_{MD,0})/d_{MD,0}$
Zn_{In}	$d_{Zn\text{-}P}$=2.3449	-0.077	0.82	2.00	2.3461	-0.076
S_P	$d_{S\text{-}In}$=2.5961	0.022	1.23	2.24	2.5977	0.022

3. Supplemental figures.

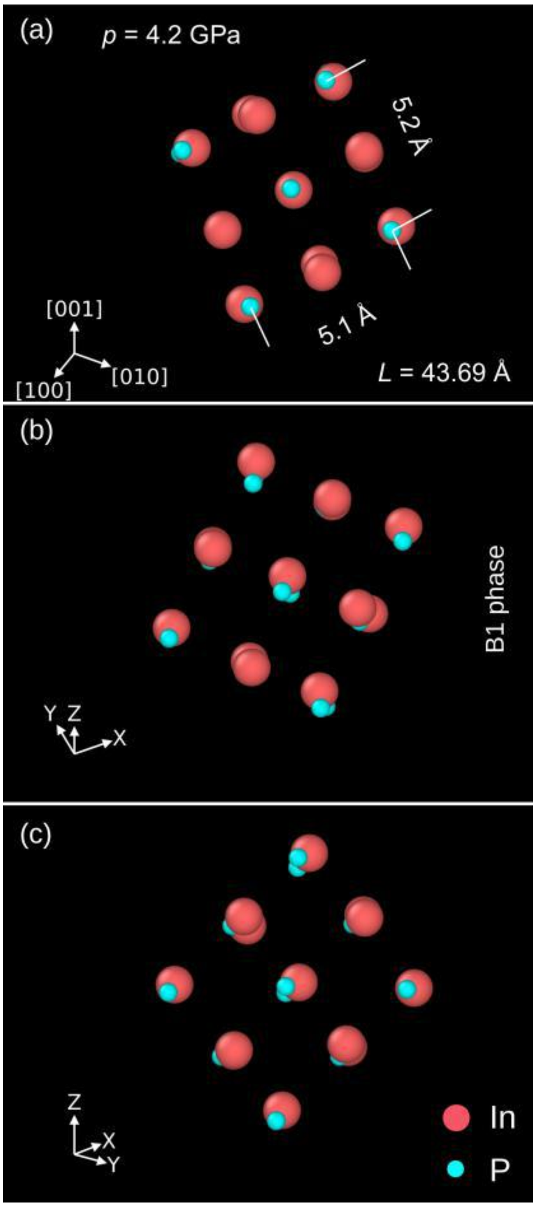

Fig. S1 Results of MD simulations. The unit cell of high-pressure phase of InP. Projections (a-c) exhibit atomic arrangements characteristic for the B1 crystal lattice.

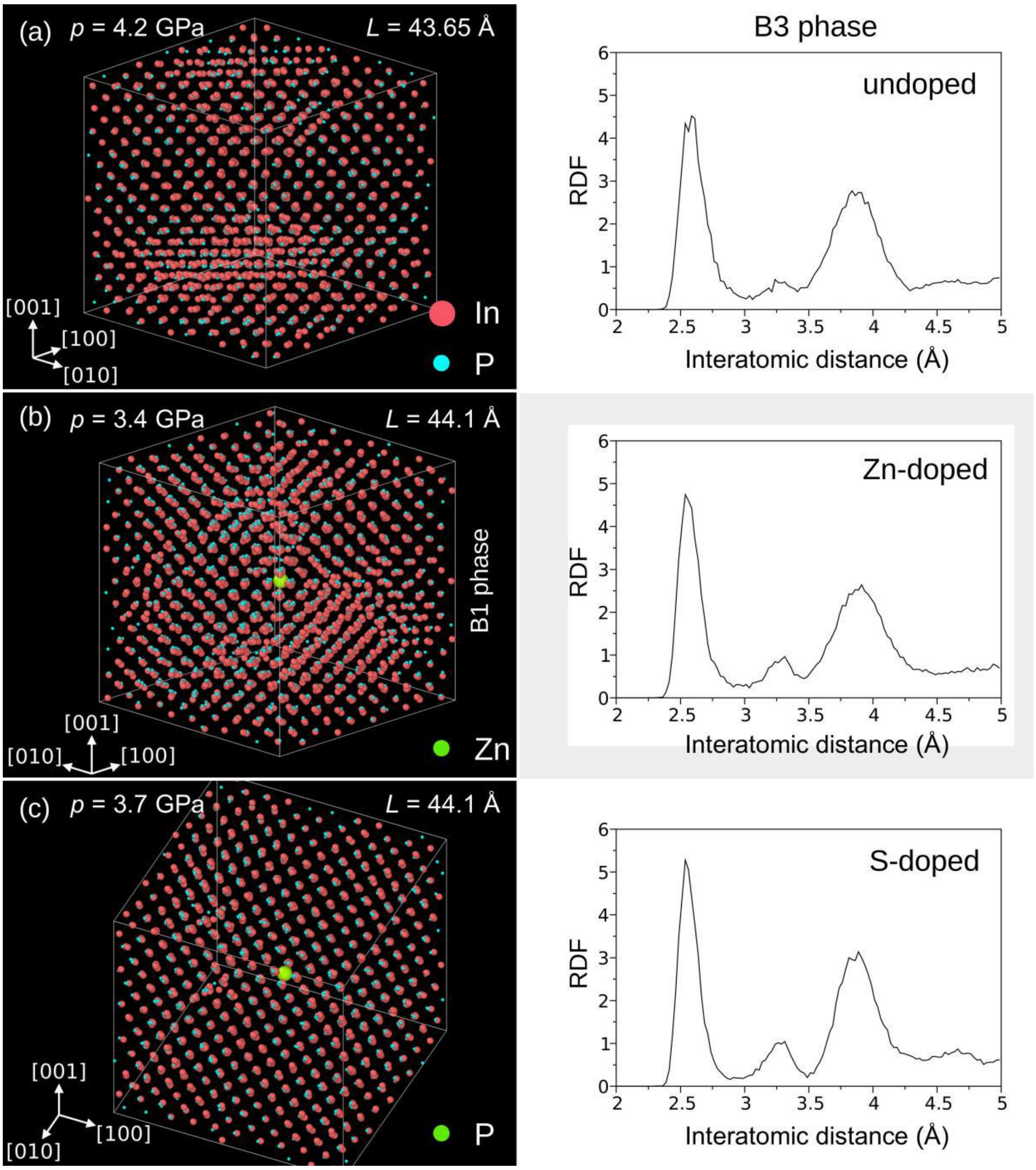

Fig. S2 Results of MD simulations. Atomic arrangement of the high-pressure phase of Zn- (a) and S-doped (b) InP crystal. Profiles of radial distribution function (RDF) convince us that the pressurized doped InP crystal exhibit the structure of B1 type as in the case of undoped InP (c).

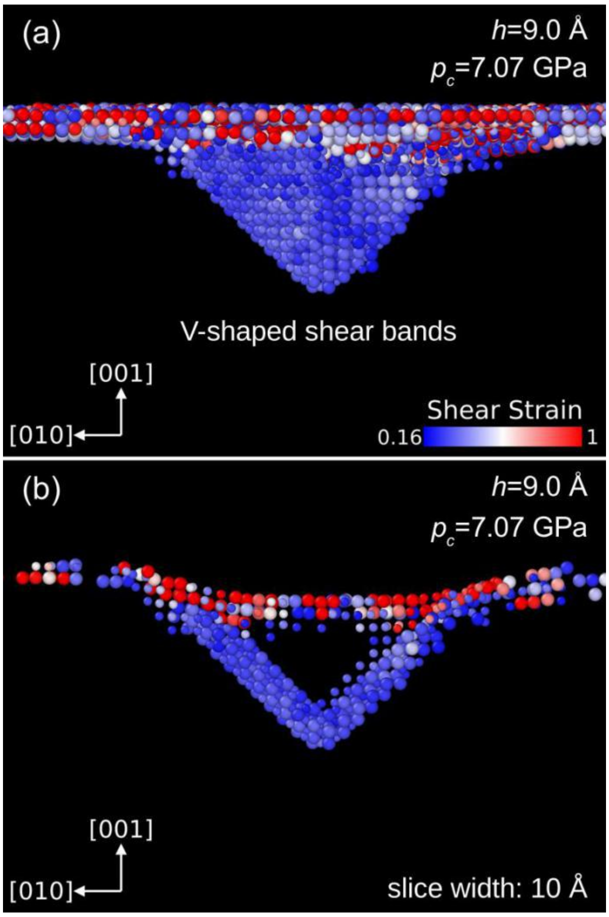

Fig. S3 Results of MD simulations. (a) The V-shaped shear bands formed during elastic deformation, directly before generation of the first dislocation. (b) Slice (width of 10 Å) showing details of the V-shaped shear bands.

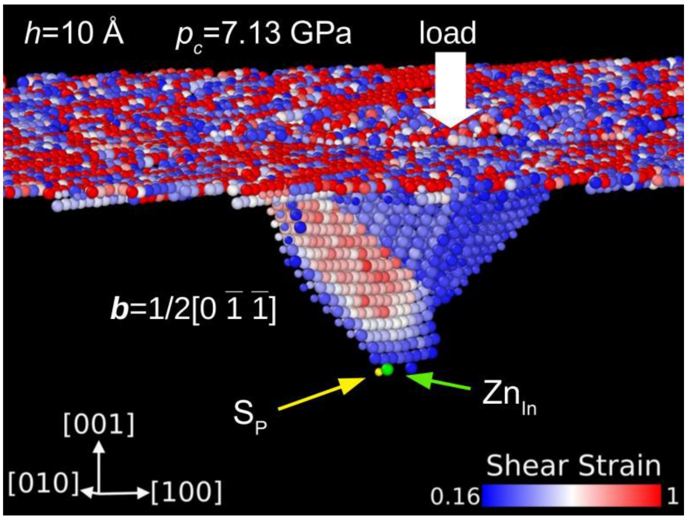

Fig. S4 Results of MD simulations. V-shaped shear bands and the first dislocation. Yellow and green atoms indicate the location of Zn and S atom, respectively, which were used for separate simulations of indentation into the doped (001) InP crystal surface.

4. Nanoindentation experiments.

Load-controlled nanoindentations (Triboindenter TI-950, Hysitron) were carried out using a conical diamond probe with a spherical tip. The experiments were performed on undoped and doped InP crystal surface of (001) crystallographic orientation, fabricated by the vertical gradient freeze method. Additional measurements of undoped and Si-doped (001) GaAs crystals were performed for the sake of comparison. Since the dislocations etch pit density was lower than 4000 cm^{-2} (approx. distance between dislocations ~150 μm), the experiments with a sharp indenter would be capable of probing both elastic and plastic properties of investigated crystals within virtually dislocation-free nanovolume. The experiments were carried out under a maximum load of 5 mN and 6 mN, for InP and GaAs, respectively. The used load function consisted of 5 s loading and equally long unloading path, with a dwell time of 2 s.

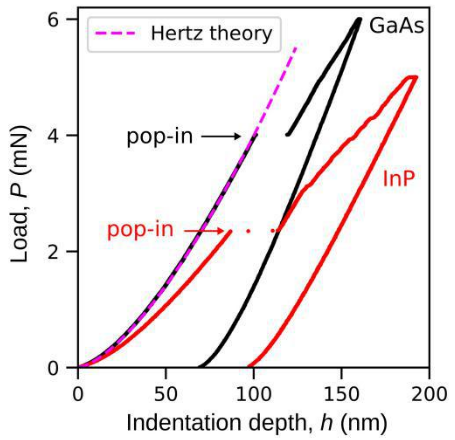

Fig. S5 Typical result of nanoindentation experiment obtained for undoped GaAs (black) and InP (red) crystals. The dashed (magenta) curve represents the Hertz fit to the *P*-*h* curve of the reference undoped GaAs crystal.

Numerous nanoindentation experiments were performed on undoped and doped InP and GaAs crystals to conclude on the origin of the their plasticity. We based the pertinent analysis of nanoindentation data on the equations derived from the Hertz theory of elastic contact between the sphere and isotropic half-space: $P(h)=(4/3)E_rR^{1/2}h^{2/3}$, and $a^2=Rh$, where: P is the indenter load, h refers to the indenter displacement, R indicates the spherical indenter tip radius, a is the radius of contact area, while E_r is the reduced Young's modulus [6]. The tip radius R=1178±12 nm was estimated using the undoped GaAs crystal by fitting the load-displacement function $P(h)$ to hundred nanoindentation P-h curves (Fig. S5). This procedure employed the reduced Young's modulus of 87.2 GPa, calculated using GaAs and the diamond elastic elastic constants [7]. Further, assuming the indenter tip radius R is known, the contact

pressure at the onset of an elastic-plastic transition (pop-in) in InP crystals was calculated as $p_c=2/3(6PE_r^2/(\pi^3R^2))^{1/3}$, where P is the load at the pop-in [6]. It is worth noting that an inaccuracy in evaluation of the contact pressure of individual pop-in results from the uncertainty of tip radius ΔR measurement by the formula $\Delta p_c=P\Delta R/(\pi R^2h)$. For example, the pop-in contact pressure of 10.7 GPa for the *P-h* curve of GaAs (Fig. S5) was determined with an accuracy of 0.1 GPa. The inaccurate estimation of load and displacement affects p_c to a lesser extent, namely at the second decimal place.

5. Lammps scripts.

Below we present the main scripts of the Lampps program used to simulate the impact of defects on the pressure of the B3→B1 phase transformation and to model the nanoindentation of (001) InP surface.

5.1 B3→B1phase transformation.

```
units  metal
boundary  p p p
variable N equal 8
variable A equal 5.87
lattice custom  $A  a1  1 0 0  a2  0 1 0  a3  0 0 1  &
       basis  0.0 0.0 0.0  basis  0.5 0.5 0.0  basis  0.5 0.0 0.5  basis  0.0 0.5 0.5  &
       basis  0.25 0.25 0.25  basis  0.75 0.75 0.25  basis  0.75 0.25 0.75  basis  0.25 0.75 0.75
region box block  0 $N 0 $N 0 $N
create_box       2  box
create_atoms  1  region  box  &
       basis  1 1  basis  2 1  basis  3 1  basis  4 1  &
       basis  5 2  basis  6 2  basis  7 2  basis  8 2
mass  1  114.818 # In
mass  2   30.973 # P
group g_box region box
pair_style vashishta
pair_coeff * * InP.vashishta In P
thermo_style custom step temp pe etotal pxx pyy pzz lx ly lz
thermo 10
velocity g_box create 300 753185
fix f1 g_box nvt temp 300 300 0.1
timestep 0.002
dump d1 all cfg 1000 ./out/relax.*.cfg mass type xs ys zs id
dump_modify d1 element In P
run 10000
undump d1
dump d1 all cfg 500 ./out/snap.*.cfg mass type xs ys zs id
dump_modify d1 element In P
fix f2 all deform 500 x scale 0.9 y scale 0.9 z scale 0.9
run 100000
```

5.2 Nanoindentation of (001) InP surface.

```
units  metal
boundary  p p f
lattice custom  5.87  a1  1 0 0  a2  0 1 0  a3  0 0 1  &
       basis  0.0 0.0 0.0  basis  0.5 0.5 0.0  basis  0.5 0.0 0.5  basis  0.0 0.5 0.5  &
       basis  0.25 0.25 0.25  basis  0.75 0.75 0.25  basis  0.75 0.25 0.75  basis  0.25 0.75 0.75
region  sim_box  block  0 70 0 70 0 70
```

```
create_box       3  sim_box
region  box  block 0 70 0 70 0 49
create_atoms  1  region  box  &
       basis  1 1  basis  2 1  basis  3 1  basis  4 1  &
       basis  5 2  basis  6 2  basis  7 2  basis  8 2
region  kula_Z  sphere  35 35 71.5 22 side in
region  kula_W  sphere  35 35 71.5 21 side out
region  pl plane 35 35 60 0 0 1 side out
region kula intersect 3 kula_Z kula_W pl
create_atoms  1  region  kula  &
       basis  1 3  basis  2 3  basis  3 3  basis  4 3  &
       basis  5 3  basis  6 3  basis  7 3  basis  8 3
region stop block 0 70 0 70 0 1 side out
region box_MD  intersect 2 box stop
mass  1  114.818
mass  2   30.973
mass  3   12.001
pair_style hybrid vashishta buck 4.0
pair_coeff * * vashishta InP.vashishta In P NULL
pair_coeff 1 3 buck 100.0 0.6 0.0
pair_coeff 2 3 buck 100.0 0.6 0.0
pair_coeff 3 3 none
dump d1 all cfg 1 ./out/reference.*.cfg mass type xs ys zs id
dump_modify d1 element In P C
run 0
group G_kula region kula
group G_box_MD region box_MD
thermo_style custom step temp pe etotal
thermo 10
velocity G_box_MD create 300 87287
fix f1 G_box_MD nvt temp 300 300 0.1
timestep 0.002
undump d1
dump d1 all cfg 20000 ./out/relax.*.cfg mass type xs ys zs fz id
dump_modify d1 element In P C
run 20000
undump d1
dump d1 all cfg 7500 ./out/l.*.cfg mass type xs ys zs fz id
dump_modify d1 element In P C
variable n loop 50
label skok
print "******** Load $n ********"
displace_atoms G_kula move 0 0 -0.5 units box
run 7500
next n
jump IN skok
```

6. References

1. P. Giannozzi et al., J. Phys.:Condens.Matter **29**, 465901 (2017).
2. A. Dal Corso, Comput. Mater. Sci. **95**, 337 (2014).
3. J. P. Perdew, K. Burke, and M. Ernzerhof, Phys. Rev. Lett. **77**, 3865 (1996).
4. J. Monkhorst and J. D. Pack, Phys. Rev. B **13**, 5188 (1976).
5. D. Chrobak, A. Chrobak, R. Nowak, AIP Advances **9**, 125323 (2019).
6. K. L. Johnson, *Contact Mechanics* (Cambridge University Press, Cambridge, 1985).
7. http://www.ioffe.ru/SVA/NSM/Semicond/InP
